# Assessing analytical methods to monitor isoAsp formation in monoclonal antibodies

**DOI:** 10.3389/fphar.2014.00087

**Published:** 2014-04-29

**Authors:** Catherine M. Eakin, Amanda Miller, Jennifer Kerr, James Kung, Alison Wallace

**Affiliations:** ^1^Department of Analytical Sciences, Amgen Inc., SeattleWA, USA; ^2^Department of Functional Biocharacterization, Amgen Inc., Thousand OaksCA, USA

**Keywords:** isoaspartic acid, hydrophobic interaction chromatography, peptide map, IdeS, fabricator

## Abstract

A ubiquitous post-translational modification observed in proteins is isomerization of aspartic acid to isoaspartic acid (isoAsp). This non-enzymatic post-translational modification occurs spontaneously in proteins and plays a role in aging, autoimmune response, cancer, neurodegeneration, and other diseases. Formation of isoAsp is also a significant issue for recombinant monoclonal antibody based protein therapeutics particularly when isomerization occurs in a complementarity-determining region due to potential impact to the clinical efficacy. Here, we present and compare three analytical methods to monitor and/or quantify isoAsp formation in a monoclonal antibody. The methods include two peptide map based technologies with quantitation from either UV integration or total ion peak areas, as well as an alternative approach using IdeS digestion to generate Fc/2 and Fab’2 regions, followed by hydrophobic interaction chromatography (HIC) to separate the population of Fab’2 containing an isoAsp. The level of isoAsp detected by the peptide map and the digested-HIC methods presented here show similar trends although sample throughput varies by method.

## INTRODUCTION

There is an increasing number of monoclonal antibody (IgG) based protein therapeutics undergoing clinical trials, or under development making them one of the fastest growing classes of protein therapeutics (Beck et al., 2008). To ensure safety and efficacy all protein therapeutics are required to be well characterized to demonstrate both process control and molecule stability. A significant concern impacting molecule stability is post-translational modifications, such as protein oxidation of methionines and/or tryptophans, deamidation of asparagines, and isomerization of aspartic acid (Asp), all of which can result in alterations to protein structure and function (Walsh and Jefferis, 2006; Schneider and Kalinke, 2008; Jefferis, 2009).

Isomerization of Asp to isoaspartic acid (isoAsp) is a non-enzymatic post-translational modification that occurs spontaneously in proteins. The isomerization process occurs via a cyclic succinimide intermediate, which undergoes hydrolysis to isoAsp and Asp typically in a 3:1 ratio. Formation of isoAsp inserts an extra methylene group into the protein backbone with a corresponding shortening of the Asp side chain by one methylene group (Geiger and Clarke, 1987; Johnson et al., 1989; Oliyai and Borchardt, 1994; Reissner and Aswad, 2003). This results in deformation in the protein structure which can impact protein function. For example, isoAsp has been postulated to play a central role in β-amyloid aggregation and neurodegenerative disorders such as Alzheimer’s disease (Roher et al., 1993a,b). Conversion of Asp to isoAsp in the complementary-determining regions (CDRs) of therapeutic antibodies has also been shown to decrease receptor binding and therefore efficacy (Cacia et al., 1996; Harris et al., 2001).

Antibody CDRs are responsible for antigen binding and are highly variable regions located in both the antibody heavy chains (HCs) and light chains (LCs). As a result of their function in antigen binding, CDR residues are located on loops that are accessible to the environment, making them susceptible to degradations including isomerization of Asp. Not surprisingly, incorporation of the extra methylene group into the antibody CDR backbone and subsequent reorientation of the side chains has been observed to impact receptor binding (Cacia et al., 1996; Harris et al., 2001; Wakankar et al., 2007a,b). Extensive studies have been performed on the kinetics of Asp isomerization and have shown that structural location in a loop as well as a glycine or a serine in the n+1 position are the most favorable for isomerization, presumably from the increased conformational flexibility afforded by the loop structure and the smaller side chain at n+1 (Geiger and Clarke, 1987; Stephenson and Clarke, 1989; Oliyai and Borchardt, 1994; Brennan and Clarke, 1995; Aswad et al., 2000; Robinson and Robinson, 2001). Although parameters including temperature and pH have been investigated and shown Asp isomerization increases in mildly acidic buffers, these buffers are the favored formulation for antibodies because other modifications including aggregation, oxidation and deamidation tend to be minimized at pH 4-6 (Shire et al., 2004; Wakankar and Borchardt, 2006).

Analytical techniques to detect and quantify isoAsp are challenging primarily because isomerization of Asp to isoAsp does not change the net charge or mass of the molecule. Despite this challenge several methods have taken advantage of the structural change associated with isomerization to separate intact antibodies with isoAsp residues from those without isomerizations including ion exchange and hydrophobic interaction chromatography (HIC; Harris et al., 2001; Wakankar et al., 2007a; Valliere-Douglass et al., 2008). However, these methods do not always provide good separation and quantitation of isoAsp particularly at low levels. Therefore, the method most frequently used to detect iosAsp in proteins is peptide mapping involving enzymatic digestion followed by reversed-phase liquid chromatography coupled to mass spectrometry. In a peptide map depending on chromatography conditions, peptides with isoAsp will often elute earlier than the unmodified peptide (Sadakane et al., 2003; Rehder et al., 2008; Ni et al., 2010). In addition, recent advances in electron transfer dissociation (ETD) mass spectrometry can be used to generate a single pair of reporter ions that are unique to isoAsp, providing an unambiguous identification of the isoAsp containing peptide (Ni et al., 2010). Although peptide mapping is the most powerful tool in isoAsp analysis it can be very time consuming and may itself actually cause sample degradations during preparation and analysis (Chelius et al., 2005). Therefore, peptide mapping may not always be practical when a large number of samples are needed for analysis. In this study, we compare three analytical methods to detect and quantify isoAsp formation. The methods include a product specific 30 min focused peptide map with UV detection, a non-product specific longer peptide map with quantitation by total ion peak area, and a new method utilizing IdeS enzyme. IdeS is an endoproteinase highly specific for a single peptide bond just below the antibody hinge region resulting in the generation of Fc/2 and the Fab’2 regions that can then be separated by HIC (Chavreux et al., 2011). Trending between the levels of isoAsp detected by these three characterization methods indicates that based on the desired throughput and accuracy analysts have multiple viable options for isoAsp quantitation.

## EXPERIMENTAL PROCEDURES

### RECOMBINANT ANTIBODIES

The monoclonal antibody used in this study was stably expressed in Chinese hamster ovary (CHO) cells and purified using conventional techniques (Shukla et al., 2007). Purified antibody was formulated in sodium acetate buffer at pH 5.2 with sorbitol.

### PROTEASE DIGESTION AND PEPTIDE MAP

Antibody was reduced and alkylated prior to peptide map analysis as described previously and digested at 37°C for 30 min in the presence of 1 M urea and a 1:10 (w/w) ratio of recombinant trypsin (Roche, Basel, Switzerland) to antibody (Valliere-Douglass et al., 2009). Peptides were separated using a Waters BEH C18 1.7 μm, 2.1 × 150 mm column at 50°C on a Waters UHPLC (Waters, Milford, MA, USA). For the focused isoAsp method the flow rate was 0.2 ml/min and the mobile phases were 0.1% TFA in water (A) and 0.1% TFA in acetonitrile (B). Peptides were eluted from the column in a linear gradient from 20 to 30% B over 15 min and were identified using a Thermo LTQ XL mass spectrometer (Thermo Scientific, Waltham, MA, USA) acquiring MS/MS spectra with collision-induced dissociation (CID) using data dependent acquisition. Quantitation of isoAsp was based on UV integration and reported as percent area of the modified peptide divided by the sum of the modified and unmodified peptides. Sample preparation and chromatography column for the non-product specific longer peptide map was as described above. Peptides were separated at a flow rate of 0.15 ml/min with 0.1% formic acid in water (A) and 0.1% formic acid in acetonitrile (B). Peptides were eluted from the column in a linear gradient from 10 to 45% B over 44 min and were identified using a Thermo Exactive Plus mass spectrometer (Thermo Scientific, Waltham, MA, USA). Quantitation of isoAsp was based on the total ion peak areas from extracted ion chromatograms of the dominant isotopic peaks from multiple charge states using commercially available software (PinPoint software, Thermo Scientific, Waltham, MA, USA). Percent isoAsp was calculated as a relative percent of the total ion area of the modified peptide divided by the sum of the total ion area from the modified plus unmodified peptides. All mass spectrometers were calibrated with commercially available calibration mix (Thermo Scientific, Waltham, MA, USA). Method robustness was evaluated using a design of experiment (DOE) based approach to determine the impact of changes to reduction, alkylation and digestion time and temperature, enzyme to substrate ratio, enzyme lot, and column lot. The method demonstrated robustness as analyzed by ANOVA using standard statistical software to ±10% of the nominal conditions. In addition, the method demonstrated specificity, linearity, repeatability, intermediate precision. The limit of detection (LOD) was calculated at 0.5 μV and the limit of quantitation (LOQ) was calculated as 0.4%.

### isoAsp IDENTIFICATION

Sample preparation and peptide map separation were carried out as described above, except the eluted peptides were split using an Advion Nanomate fraction collection robot (Advion Biosciences, Ithica, NY, USA). Briefly, the flow was split and 150 nL was analyzed on-line with a Thermo LTQ XL mass spectrometer with ETD capability (Thermo Scientific, Waltham, MA, USA). The remaining volume was collected in a 96-well plate for off-line analysis. The peptide containing isoAsp and the non-isomerized peptide were analyzed by MS using the Nanomate in static-nanospray infusion mode using ETD fragmentation with supplemental activation.

### HYDROPHOBIC INTERACTION CHROMATOGRAPHY

Isolation of stressed antibody containing ~40% isoAsp was performed using two Dionex ProPac HIC-10 7.8 × 75 mm HIC columns in series (Dionex, Sunnyvalem, CA, USA). Samples were separated at a flow rate of 0.5 ml/min on an Agilent 1100 HPLC (Agilent Technologies, Santa Clara, CA, USA). The mobile phases for separation were 1 M ammonium sulfate 10 mM acetate pH 5.2 (A) and 10 mM acetate pH 5.2 (B). 50 μg of sample mixed 1:1 with mobile phase A was bound to the column equilibrated in 100% mobile phase A. Samples were eluted in a linear gradient of 40–60% B over 40 min. The separation was monitored by absorbance at 280 nm. Peaks fractionated from HIC for subsequent analysis were immediately buffer exchanged into sodium acetate buffer at pH 5.2 with sucrose. Analytical HIC separation was performed using two Dionex ProPac HIC-10 4.6 × 100 mm HIC columns in series (Dionex, Sunnyvalem, CA, USA). Samples were separated on a Waters Alliance HPLC (Waters, Milford, MA, USA) using the mobile phases, flow rate and gradient described above.

### PROTEASE DIGESTION AND HIC

Sixty micrograms of antibody was digested into the Fc/2 and Fab’2 by 60 U of IdeS (Genovis, Lund, Sweden) at 37°C for 30 min (Chavreux et al., 2011). Digestions were carried out in 50 mM sodium phosphate, 150 mM sodium chloride pH 6.6. Following digestion samples were analyzed by HIC as above. Method robustness was evaluated using a DOE based approach to determine the impact of changes to digestion buffer pH, digestion time, digestion temperature, reduction buffer concentration, reduction temperature, and reduction time. The method demonstrated robustness as analyzed by ANOVA using standard statistical software to ±10% of the nominal conditions for all parameters except digestion temperature. At 5°C under the nominal digestion temperature the level of undigested material increased by 2.5% indicating control of digestion temperature is needed for digestion efficiency. In addition, the method demonstrated specificity, retention time repeatability and accuracy. The LOD was calculated at 0.6 μV and the LOQ was calculated as 0.2%.

### INTACT MASS

Hydrophobic interaction chromatography fractions were desalted prior to mass analysis by analytical size exclusion chromatography (SEC) coupled to an electrospray ionization (ESI) source TOF/MS (Brady et al., 2008). Briefly, 10 μg of sample was injected onto a BEH 1.7 μm, 4.6 × 150 mm SEC column (Waters, Milford, MA, USA). Samples were eluted isocraticly in 15% acetonitrile, 0.1% formic acid at a flow rate of 0.4 mL/min directly into an Agilent TOF/MS (Agilent, Santa Clara, CA, USA). Raw MS data was deconvoluted with MassHunter Qualitative Analysis software.

### RECEPTOR BINDING

The antibody used in this study was developed to target a cell surface receptor; therefore, a cell based binding assay was used to report on antibody receptor binding and potency. CHO cells were stably integrated with the antibody receptor in-house and the stable clone expressing high levels of the receptor was produced. A clone demonstrating both a high level of receptor expression and a strong binding to the native ligand were used for all receptor binding experiments. The cells were maintained in DMEM media with Glutamax (Invitrogen, Carlsbad, CA, USA) containing 10% FBS, non-essential amino acids, Pen-Strep-L-Glut, and Hygromycin B at 37°C with 5% CO_2_ in a humidified incubator. 24 h prior to the experiment cells were transferred into DMEM described above without Hygromycin B. To measure receptor binding cells are incubated with a fixed concentration of biotin-labeled native ligand and a varying concentration of antibody reference standard and test samples. Binding of the biotin-labeled ligand to the receptor expressed on the cells is detected by the addition of phycoerythrin conjugated to streptavidin. After washing of unbound ligand the fluorescence response of phycoerythrin is measured and plotted as a function of log dose for both reference standard and test samples. Effective competition of the test samples for receptor binding produced a decrease in fluorescence signal. Test sample activity is determined by comparing the response of the test samples to that of the reference standard.

## RESULTS

### CHARACTERIZATION OF isoAsp IN STABILITY SAMPLES

**FIGURE 1:**
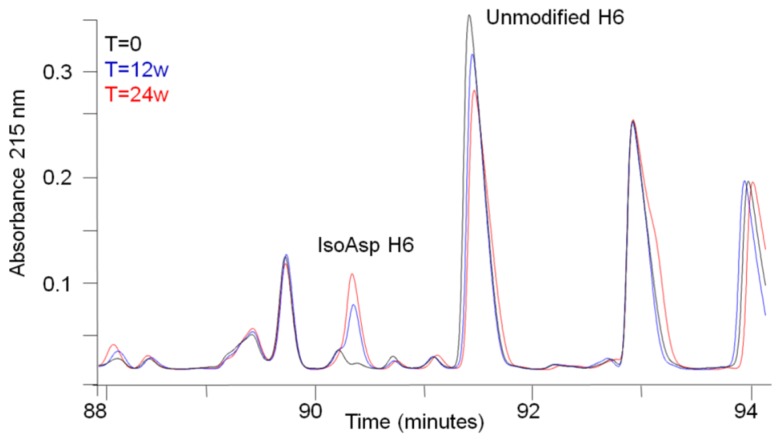
**Peptide map analysis of stability samples.** Selected region of tryptic peptide maps of a stability sample held at 40°C for 0 weeks (black), 12 weeks (blue), or 24 weeks (red). The peptide corresponding to isoAsp H6 and unmodified H6 are labeled.

During stability studies of this monoclonal antibody, few chemical modifications were observed. However, tryptic peptide map analysis after long-term storage at elevated temperature in a mildly acidic formulation buffer revealed the increase of a new peak eluting ~1 min before the H6 peptide (**Figure 1**). This peak was determined by mass measurements and MS/MS sequencing using CID to have the same molecular weight and sequence as the H6 peptide. Isomerization of Asp to isoAsp is known to cause a shift in peptide retention time without changing the molecular mass. In addition, the residue after Asp in the H6 peptide is a glycine, which kinetic studies have consistently shown to be favorable for Asp isomerization (Oliyai and Borchardt, 1994; Aswad et al., 2000). Therefore, isomerization of Asp to isoAsp in the H6 peptide is the suspected chemical modification for the new species eluting prior to the H6 peptide.

**FIGURE 2:**
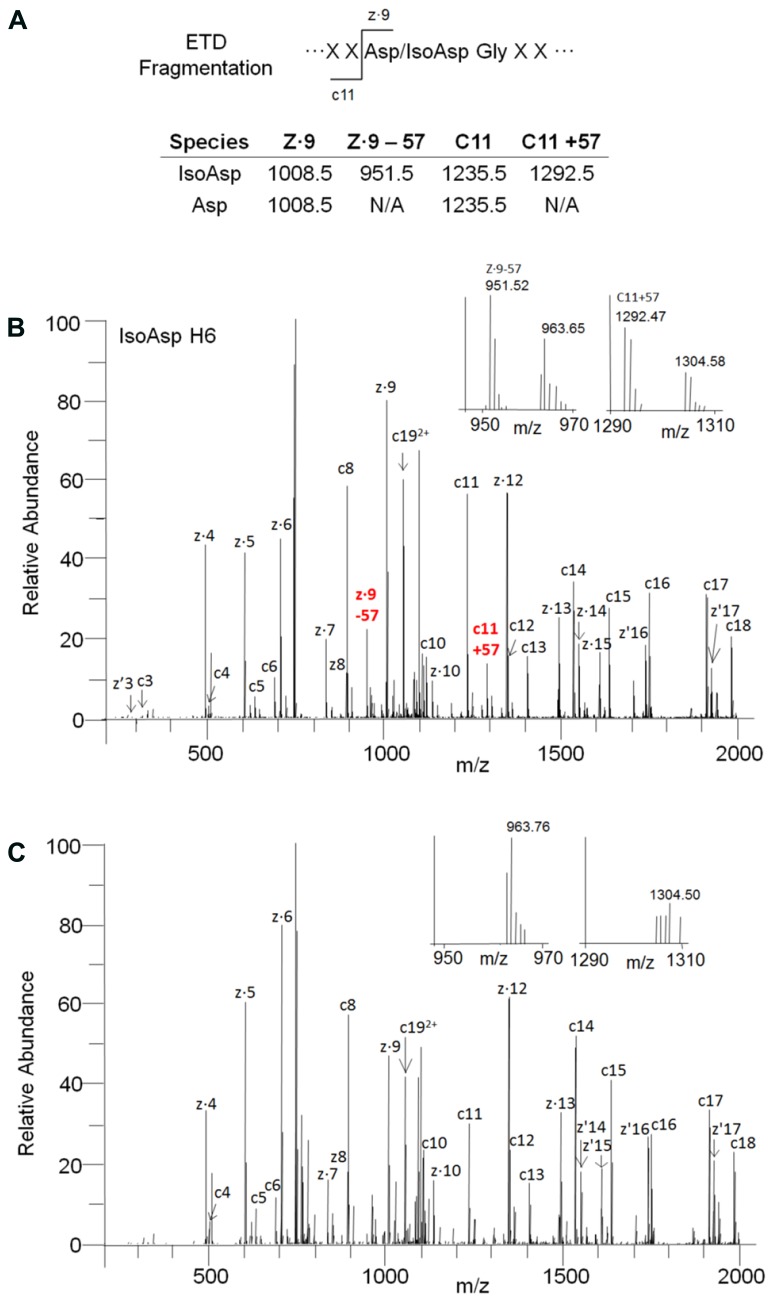
**Identification of isoAsp55 in stability samples. (A)** Observed masses for ETD fragmentation of isomerized and unisomerized H6 peptide. **(B)** ETD fragmentation pattern for the isoAsp H6 peptide. The inset is a zoomed view showing the reporter ions at 951.5 (z.9–57 Da) and 1292.5 Da (c11 + 57 Da). **(C)** ETD fragmentation pattern for the H6 peptide. The inset is a zoomed view showing the lack of reporter ions at 951.5 Da and 1292.5 Da.

The identity of the new species was confirmed to be isoAsp in the H6 peptide by ETD fragmentation mass spectrometry with supplemental activation. Although isoAsp formation results in incorporation of an extra methylene group in the protein backbone the fragmentation pattern and masses observed during CID are the same for isoAsp and Asp due to the corresponding loss of a methylene from the Asp side chain. Recent advances in ETD mass spectrometry have shown that ETD can generate a single pair of reporter ions (c + 57 and z - 57) that are unique to isoAsp allowing unambiguous assignment of isoAsp containing peptides (Chan et al., 2010; Ni et al., 2010). The ETD fragmentation pattern of the peptide eluting prior to H6 clearly produced ions corresponding to c11 + 57 Da (1292.5 Da) and z9–57 Da (951.5 Da) which indicated the presence of isoAsp at Asp 55 in the HC. These ions were not detected in ETD fragmentation of the H6 peptide (**Figure 2**). The peptide map elution profile coupled with the accurate mass measurement and the ETD fragmentation pattern confirmed the chemical modification on the species eluting prior to the H6 peptide is indeed the H6 peptide with an isoAsp at position 55. Asp55 is located on the HC CDR2, therefore, isomerization to isoAsp may have an impact on receptor binding and molecule potency.

**FIGURE 3:**
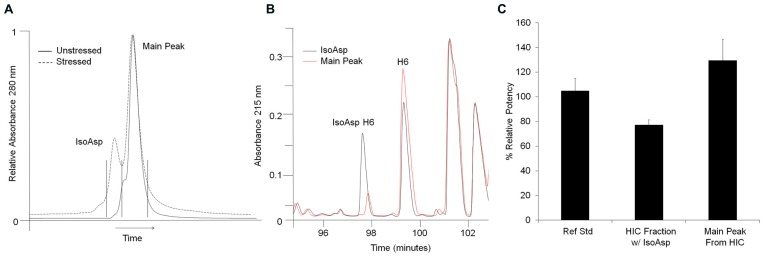
**Isolation and potency evaluation of isoAsp containing antibody. (A)** HIC separation of isoAsp from main peak in stressed (dashed) material. Unstressed material is shown for reference (solid). Stressed material was collected in two fractions indicated by the vertical black lines. The x-axis has been normalized to the main peak to account for normal chromatographic drift. **(B)** Selected region of the peptide maps of HIC fractionated material. The isoAsp peak is shown in black and the main peak from stressed material in red. **(C)** Percent relative potency of isoAsp antibody and main peak fractions collected from HIC of stressed antibody. Potency levels are set relative to the reference standard control.

Potency evaluation in an *in vitro* cell based receptor binding assay found that isomerization of Asp55 decreased receptor binding compared to unisomerized antibody. HIC fractionation of a stability sample stressed for 24 weeks at 40°C was used to separate unisomerized from isoAsp containing antibody (**Figure 3A**). Peptide map analysis of the two distinct HIC fractions found that the earlier eluting peak had 40% isoAsp H6 peptide, while the main peak contained 7% isoAsp. The presence of 40% isoAsp H6 in the earlier eluting HIC peak suggested that this species contained an isoAsp in only one of the two antibody HCs (**Figure 3B**). Potency testing of the two HIC fractions found that relative to the reference standard, the HIC fraction containing one isoAsp55 had a 22% decrease in potency, while the main peak fraction isolated under the same conditions had a 31% increase (**Figure 3C**). The apparent increase in potency of the main peak relative to the reference standard could be from the removal of other covalent modifications or high molecular weight material during fractionation. Cell based assays inherently have higher variability than other analytical assays. With the typical precision in the potency assay being about ±10%, the decrease in potency of the isoAsp containing material may therefore be at the edge of a significant change in potency. However, the 53% delta between the isoAsp containing species and main peaks may suggest that the chemical modification of Asp55 to isoAsp in the CDR2 has an impact on receptor binding and could potentially impact molecule efficacy.

### HIC ANALYSIS OF STABILITY SAMPLES

**FIGURE 4:**
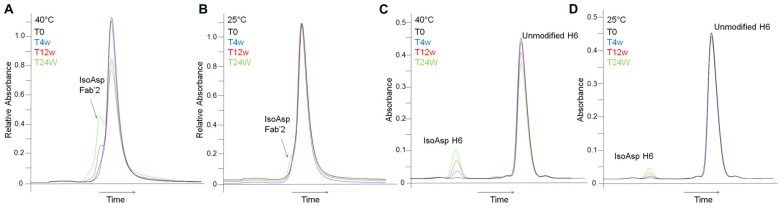
**Detection of isoAsp by intact HIC separation and focused peptide map.** HIC of stability samples held at 40°C **(A)** or at 25°C **(B)**. A214 nm trace from the focused peptide map of stability samples held at 40°C **(C)** or at 25°C **(D)**. *T* = 0 weeks (black), *T* = 4 weeks (blue), *T* = 12 weeks (red), *T* = 24 weeks (green). The x-axis has been normalized to the main peak to account for normal chromatographic drift.

The potential impact of isoAsp to potency indicated that the conversion of Asp55 to isoAsp should be monitored during development and potentially during long term storage. As a higher throughput alternative to peptide mapping intact HIC was explored as a characterization method to monitor isoAsp content. HIC has previously been used to separate populations of antibody which are covalently modified during stability programs, including separation of succinimide intermediates from unmodified antibodies (Valliere-Douglass et al., 2008). Separation of isoAsp from non-isomerized antibody can be achieved by HIC, however, the separation between the two species is not baseline resolved making quantitation difficult. In addition, samples held at 25°C for 12 weeks and 24 weeks have 6.8 and 12.3% isoAsp antibody, as determined by peptide mapping; however, at these levels the isoAsp species appears as an early eluting shoulder off of the main HIC peak which cannot be integrated (**Figure 4**). Conversion to isoAsp at 4°C is much slower than at elevated temperatures with samples increasing by 0.5% after 6 months of storage. This indicates that although formation of isoAsp is slower at 4°C the level is increasing at recommended storage and the HIC method does not provide sufficient resolution to monitor this change.

### DIGESTED-HIC ANALYSIS OF STABILITY SAMPLES AND CORRELATION TO PEPTIDE MAPS

**FIGURE 5:**
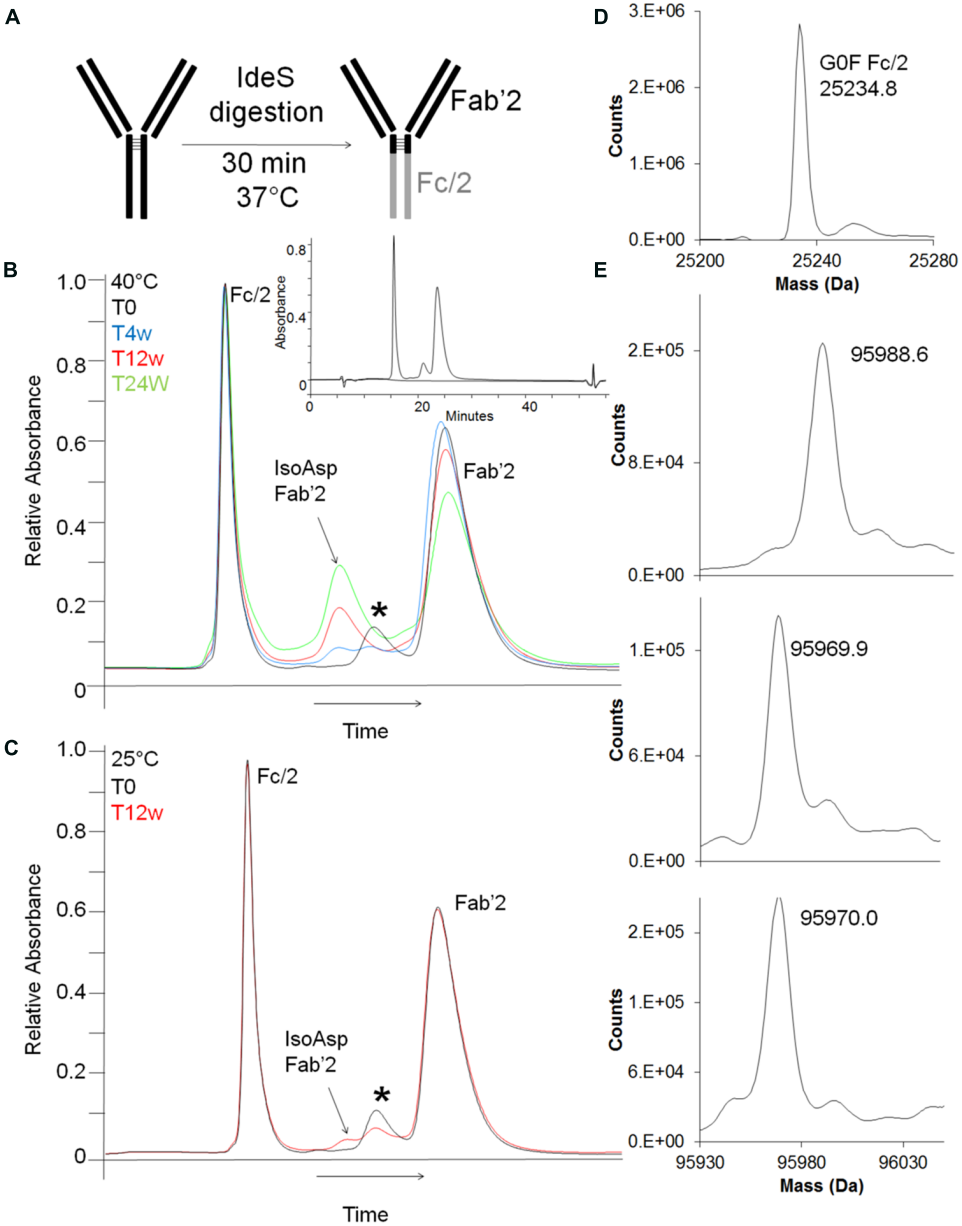
**Detection and characterization of isoAsp by digested-HIC. (A)** Shematic representation of antibody digestion by IdeS protease. **(B,C)** HIC separation of stability samples held at 40°C **(B)** or at 25°C **(C)** after digestion with IdeS. *T* = 0 weeks (black), *T* = 4 weeks (blue), *T* = 12 weeks (red), *T* = 24 weeks (green). The peak labeled with * is Fab’2 with one HC containing an unclyclized N-terminus. Inset shows full scale view of digest-HIC method (blue) overlaid with a blank (black). The x-axis has been normalized to the main peak to account for normal chromatographic drift. Peak identifications shown in **(B,C)** were assigned by intact mass analysis **(D,E)** of peaks fractionated from HIC. **(D)** Intact mass of the Fc/2 fractionated from the HIC. **(E)** Top panel is Fab’2 with one HC not cyclized to pyroglutamic acid and corresponds to the peak labeled with * in **(B,C)**. The middle panel corresponds to the Fab’2 with isoAsp in one HC and the bottom panel is Fab’2.

Better chromatographic separation between isoAsp and unisomerized antibody was achieved by digested-HIC, where proteolysis is carried out under native conditions followed by HIC separation. The IdeS endoproteinase cleaves IgG2 antibodies between the alanine and the glycine of the PPVAG sequence in the HC CH2 domain near the hinge region generating two fragments, a Fc/2 and a Fab’2 (**Figure 5A**). Digestion with IdeS occurs under native conditions allowing HIC separation to take advantage of the structural changes associated with isoAsp to separate Fc/2, isoAsp-Fab’2 and Fab’2. Digested-HIC analysis of stability samples revealed four peaks, one of which increased and two of which decreased with time (**Figures 5B,C**). To further characterize these four peaks they were fractionated from the HIC and identified by intact mass. The molecular masses of the Fc/2 and Fab’2 were 25234.8 Da and 95970.0 Da, respectively. These values are within 20 and 13 ppm of the theoretical masses for Fc/2 (25234.3 Da) and Fab’2 (95971.2 Da). The N-terminal residue is a glutamine (Q), which cyclizes to pyroglutamic acid (pE) during production; however, it is not uncommon to have incomplete cyclization. The peak eluting immediately before the Fab’2 that decreases with time has a mass of 95988.6 Da. This matches within 11 ppm the expected mass of 95988.2 Da for Fab’2 with one Q-HC and one pE-HC. The decrease in this peak in stability samples over time is a result of forced cyclization under storage conditions and is consistent with observations for other antibodies (Rehder et al., 2008). The peak that increased with time between the Fc/2 and partially cyclized Fab’2 was identified as Fab’2 with one isoAsp HC. The molecular weight of this species is 95969.0 which is 23 ppm from the expected mass of 95971.2 Da for the Fab’2 (**Figures 5B–E**). The shift in HIC retention time but the same molecular mass as the Fab’2, as well as an increase during storage that correlated well with a decrease in Fab’2 signal all suggest that this peak is iso-Asp Fab’2. In addition, peptide map analysis of this HIC fraction confirmed this species as Fab’2 with one isoAsp at Asp55 in the HC based on chromatographic retention time and peptide mass (data not shown). Digestion of the antibody into two smaller fragments prior to HIC increased the resolution between the isoAsp and unisomerized species. With a LOQ for this characterization assay of 0.2% this allowed for easy integration of isoAsp levels even in un-aged samples with ~1% or less isoAsp.

**FIGURE 6:**
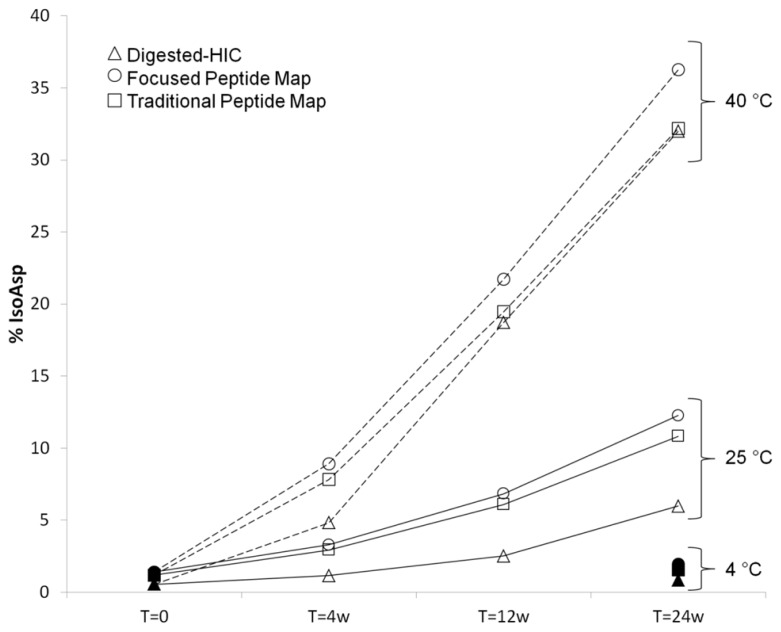
**Comparison of percent isoAsp determined by digested-HIC (Δ), UV integration from a focused peptide map (◯), and total ion area from a longer traditional peptide map (□).** Samples were held at 4°C (filled symbols), 25°C (solid line), or 40°C (dashed line) for up to 24 weeks. Samples at 4°C for 4 weeks and 12 weeks were not tested due to the slow rate of isoAsp formation at this temperature.

The percent of isoAsp as calculated by digested-HIC had a similar trend to the levels calculated from a focused peptide map using UV integration and a non-product specific peptide map with quantitation from total ion area (**Figure 6**). The focused peptide map was developed specifically for this antibody to separate the peptides containing isomerized Asp55 and unisomerized Asp55. The main method deliverables included baseline resolution between the two species to allow for easy UV integration, minimal co-elution with other species and a total chromatography time of 30 min or less (**Figures 4C,D**). The traditional peptide map is applicable to multiple antibodies and utilizes a longer gradient of 80 min plus an additional gradient to minimize carry over between each sample for a total run time of 140 min. A quantitation strategy for the non-product specific peptide map utilizing total ion area rather than a UV trace still necessitates the need for chromatographic resolution between the isomerized and unisomerized species but not from other co-eluting species of different mass. Peptide mapping is conducted under reducing conditions, whereas the digest-HIC method is non-reducing. Therefore to account for the presence of two H6 peptides in each Fab’2 the percent isoAsp calculated from the peptides maps is doubled. The percent isoAsp calculated from digested-HIC and total ion area are lower than the level calculated by UV integration from the focused peptide mapping. This trend is observed across all samples tested indicating a consistent offset between the methods. The exact cause for these discrepancies is unknown, but one potential explanation might be a lower than expected recovery from either the IdeS digestion or the HIC chromatography or coelution of other species in the focused peptide map. In the digested-HIC method Fab’2 with one isoAsp HC is observed, but Fab’2 with two isoAsp HCs is not observed. Fab’2 with two isoAsp HCs could possibly co-elute from the HIC with the Fc/2; however, the Fc/2 percent area is unchanged in all of the stability samples and there was no evidence by intact mass for Fab’2 in the Fc/2 fraction. Alternatively, Fab’2 with one and two isoAsp could possibly co-elute. Regardless, the overall trend in isoAsp content is similar between the three methods.

## DISCUSSION

Detailed characterization of protein therapeutics is central to ensuring they are both safe and efficacious for patients. Among the post-translational modifications that can impact protein function is isomerization of Asp to isoAsp (Cacia et al., 1996). Here, we evaluate three characterization methods with varying degrees of throughput for detection and quantification of isoAsp in antibodies. The methods include two peptide map based methods with longer sample preparation times but different chromatographies and a new method that utilizes specific digestion of the antibody with IdeS into Fc/2 and Fab’2 fragments followed by HIC separation. A correlation between the levels of isoAsp detected in the peptide maps and the digested-HIC method suggest that the digested-HIC method could be routinely used to monitor Asp isomerization as a higher throughput alternative to peptide mapping, particularly for monitoring trends in Asp isomerization.

Peptide mapping coupled with mass spectrometry is the single most powerful analytical technique for detection and quantitation of isoAsp species. However, owing to what can be the time consuming nature of peptide mapping there is a need for higher throughput methods capable of monitoring and quantifying isoAsp levels, particularly for the large sample sets often associated with antibody stability studies. As a characterization method for analyzing isoAsp content in antibodies with shorter sample preparation time, we have utilized the IdeS protease to specifically cleave antibodies into Fc/2 and Fab’2 under native conditions followed by HIC separation. Breaking the intact antibody into two smaller domains affords better chromatographic separation and therefore quantitation of isoAsp containing species particularly at low levels. Separation by HIC is dependent on differences in protein hydrophobicity. A central feature of the digested-HIC method is the non-denaturing aspect of the IdeS digestion which allows HIC separation to take advantage of the structural changes associated with isoAsp formation. The estimated time required for the digested-HIC method is less than 1.5 h, which includes 30 min for sample digestion, 40 min for HIC chromatography and ~5 min for data analysis. This compares favorably against the approximately 2.5 h needed for sample preparation alone in the peptide map methods (**Table 1**). In the focused peptide map the total chromatography time is less than a quarter that of the non-product specific peptide map, which increases sample throughput significantly. However, by focusing the map for isoAsp55 quantitation the ability to monitor other product quality attributes within the same method is lost. One of the greatest advantages of the traditional peptide map is the potential to monitor more than one attribute and arguably this might be enough to offset the increased time required for this method.

**Table 1:** Evaluation of isoAsp detection methods.

Method	Sample prep time (min)	Chromatography time (min)	Analysis of isoD only (min)	Location specific information
Peptide map	150	140^*^	10	Yes
Focused peptide map	150	30	5	Yes
Digested-HIC	30	40	5	Domain specific

* Time includes separation gradient and a required cleaning gradient between each sample.

There is a difference in the absolute percent of isoAsp determined from the digested-HIC method introduced here and the two peptide map methods. There is also a small difference in isoAsp quantitation between the two peptide maps based methods; however, in all cases the trend in isoAsp formation is the same. For all samples, the level of isoAsp determined by the digested-HIC method is lower than that determined by peptide mapping. The discrepancy between the methods is consistent across multiple samples suggesting method variability is not solely accountable for the observed difference. The decrease in the percent isoAsp detected by HIC could be the result of poor recovery or other undetected modifications that offset the impact of isoAsp to the HIC. Alternatively, the focused peptide map could be over reporting the level of isoAsp H6. The percent isoAsp from the peptide maps was calculated based on UV integration of only the isomerized H6 peptide and the unisomerized H6 peptide. Therefore, co-eluting species or other modifications in the isoAsp containing peptide could be inflating the percent isoAsp. This is likely the case in the 40°C stress samples where the discrepancy between the two peptide map methods increases with time (**Figure 6**). Quantitation of isoAsp from the non-product specific peptide map and total ion area resulted in a decrease in the percent of isoAsp relative to that calculated by UV. However, the decrease did not account for the complete discrepancy between the HIC and focused peptide map method. Even with a difference in the absolute value, the results presented here indicate that a similar trend in percent isoAsp is observed between the methods and suggests that if needed alternative approaches can be utilized to monitor Asp isomerization during characterization.

Isomerization of Asp in proteins has been observed to occur *in vivo* in a variety of pathways including aging, neurodegenerative disorders, regulation of apoptosis, and autoimmune responses (Roher et al., 1993a; Robinson and Robinson, 2001; Doyle et al., 2007; Cimmino et al., 2008). In naturally occurring antibodies the impact of a CDR Asp isomerizing to isoAsp is most likely negligible owing to the diverse assembly of sequences with varying affinity associated with polyclonal antibodies. Thus, there is likely little to no evolutionary advantage to the organism to select against CDR sequences prone to isoAsp despite the structural impact of isomerization. In stark contrast are monoclonal antibodies used as protein therapeutics. These antibodies have very specific targets with commensurate impact to the binding affinity between the antibody and target resulting in a significant consequence to drug efficacy. Previous studies have observed that a single isomerization event in either antibody LC or HC CDRs greatly reduces binding activity (Cacia et al., 1996; Rehder et al., 2008; Yan et al., 2009). Further studies have also shown that isomerization of antibodies in *in vivo* animal models can trigger a spontaneous degradation pathway for antibody clearance (Huang et al., 2005). Our *in vitro* studies are in agreement with these previous results and revealed a potency difference between material with isoAsp in the HC CDR2 and unisomerized protein, demonstrating again the significant impact a post-translational modification at a single site can have on protein function. Previous efforts have been undertaken to evaluate modifications to the CDRs that would prevent isomerization either by mutation of the isomerizing Asp or mutation of the n+1 amino acid, in both cases receptor affinity was negatively impacted highlighting the sensitivity of antigen recognition to subtle CDR sequence changes (Presta et al., 1993). These studies have demonstrated that Asp residues prone to isomerization cannot simply be engineered out of therapeutic antibodies; therefore, efficient methods for isoAsp detection and quantitation are necessary.

## AUTHOR CONTRIBUTIONS

Catherine M. Eakin designed the experiments; Catherine M. Eakin, Amanda Miller, Jennifer Kerr and James Kung performed the experiments; Catherine M. Eakin, Amanda Miller, Jennifer Kerr and James Kung performed data analysis; Alison Wallace provided scientific expertise; Catherine M. Eakin wrote the manuscript.

## Conflict of Interest Statement

The authors declare that the research was conducted in the absence of any commercial or financial relationships that could be construed as a potential conflict of interest.
